# Medical education and population health—A framework in the design of a new undergraduate program

**DOI:** 10.3389/fpubh.2022.1068092

**Published:** 2022-12-07

**Authors:** Robert W. Armstrong, Michaela Mantel, Gijs Walraven, Lukoye Atwoli, Anthony K. Ngugi

**Affiliations:** ^1^Medical College, Aga Khan University, Nairobi, Kenya; ^2^Department of Pediatrics, The University of British Columbia, Vancouver, BC, Canada; ^3^The Health Associates Ltd, Berlin, Germany; ^4^Aga Khan Development Network, Geneva, Switzerland; ^5^Department of Medicine, Brain and Mind Institute, Aga Khan University, Nairobi, Kenya; ^6^Department of Population Health, Aga Khan University, Nairobi, Kenya

**Keywords:** medical education, population health, health science education, health determinants, developing countries, public health, global health

## Abstract

Health sciences curricular planners are challenged to add new content to established education programs. There is increasing pressure for content in public health, health systems, global health, and planetary health. These important areas often compete for curricular time. What is needed is a convergence model that builds a common framework within which students can integrate areas and better align this knowledge to the individual client or patient who they have responsibility to support. A population health framework is proposed for health sciences education programs that supports a common conceptual understanding of population health. The framework links five thematic areas that have influence on health and wellbeing and a sixth element that defines the range of methodologies essential to understanding health and wellbeing, from the individual to the population. The five areas providing convergence are: (1) the biopsychosocial development of the individual, (2) the socioeconomic factors that influence health and wellbeing, (3) the physical natural and built environment including climate, (4) the continuum of public health and health care systems, and (5) the nation state and global relationships. Using this framework, students are encouraged to think and understand individual health and wellbeing in context to the population and to utilize the appropriate methodological tools to explore these relationships. Planning for a new undergraduate medicine program illustrates the curricular elements that will be used to support student learning with foundation knowledge applied and tracked throughout the program. The proposed framework has application across health sciences disciplines and serves to build a common understanding that supports cross professional communication and collaboration.

## Background

Starting a new undergraduate medical program creates opportunity for providers to assess the frameworks used for structuring population health education. The Aga Khan University Medical College in East Africa had this opportunity when planning for an undergraduate medical program in Nairobi, Kenya.

The Aga Khan University (AKU) is a private not-for-profit university with a strong development mandate and is one of 10 agencies of the Aga Khan Development Network (AKDN) (https://www.akdn.org/). The university has campuses in Pakistan, Afghanistan, United Kingdom, and the East African countries of Kenya, Tanzania, and Uganda (https://www.aku.edu/). The first health sciences campus was established on a greenfield site 40 years ago in Karachi, Pakistan where there is now a well-established tertiary teaching hospital with undergraduate and postgraduate medical and nursing/midwifery schools.

AKU launched a new academic medical center in East Africa through conversion of an existing community hospital and establishment of a Faculty of Health Sciences based in Nairobi, with campuses also in Kampala, Uganda, and Dar es Salaam, Tanzania. The School of Nursing and Midwifery was first to be established in 2002 followed by the Medical College and postgraduate medical programs, with the goal of transforming the Nairobi community hospital to a university hospital and building faculty from graduates of the residency programs in advance of starting the undergraduate program.

Over several years, the Nairobi-based AKU tertiary teaching hospital has matured, achieving Joint Commission International accreditation in 2013. The medical college has been established with nine core residency programs and several fellowship programs. In Dar es Salaam five new residency programs have started and a university campus is under development in Kampala which will support nursing and medical training in a new university hospital. The AKU health system is complemented by four Aga Khan Health Services (AKHS) hospitals (Mombasa and Kisumu in Kenya and Dar es Salaam and Mwanza in Tanzania) and in combination with AKU there are over 100 community-based health centers in the three countries. As development focused not-for-profit organizations, AKU and AKHS collaborate with the public and private health services and universities to advance clinical practice, education and research that supports health and health care of the populations. In this context, AKU is committed to the inclusion of population health as a core foundation of the undergraduate medical program with the goal that graduates will have the knowledge and skills to contribute to addressing the significant health challenges of the East African Community in the twenty-first century. The start of the undergraduate medicine program was delayed due to the COVID-19 pandemic but is now scheduled to begin in September of 2023.

A Department of Population Health was established in the AKU Medical College in 2016 with a mandate to develop the framework that would structure an approach to population health education and advance population health research. Planning began with an internal “thinking group” and a survey of population health education in the context of East Africa (1). There was a consensus that a strong focus on population health within the undergraduate curriculum can potentially contribute to a more capable health workforce that is better aligned to the needs of the region and would generate leaders with national and regional impact. The Population Health Department will support both medicine, nursing, and allied health sciences education within the Faculty of Health Sciences. This paper provides the framework used for defining population health and demonstrates how it will be integrated into the new undergraduate medical education program.

## The population health framework

Curricular planning in medical education is increasingly focused on pedagogical approaches that integrate content across disciplines with designs that connect basic science knowledge to the clinical case experience of students. Planners are challenged to add new content to a program that is already information dense and constantly in need of updating. Attention to reform has primarily been directed to integrating the biomedical sciences. However, there is recognition that students of the twenty-first century need a broader and more extensive set of skills in the areas of public health, health systems, global health, and planetary health. These important areas often compete for curricular time and are often proposed as distinct units within the curriculum (2–4).

What is needed is a convergence model of population health that builds a common framework within which students can integrate areas and better align this knowledge to the individual client or patient who they have responsibility to support (5, 6). Population health is by no means a “new” concept but the framing of population health within health professional curricula has had limited attention.

Geoffrey Rose, a clinical epidemiologist, advanced the concept of population health as an important framework “bridging clinical medicine with its focus on individuals, and epidemiology and public health, with their focus on populations.” His pioneering paper “Sick Individuals and Sick Populations” challenged the historical separation of these fields (7). As Rose (7) noted “it is an integral part of good doctoring to ask not only, ‘What is the diagnosis, and what is the treatment’ but also ‘Why did this happen, and could it have been prevented?”’

There has been extensive discussion regarding the definition of population health (8–14). Kindig and Stoddart (9) define population health as “the health outcomes of a group of individuals, including the distribution of such outcomes within the group” and note that “in addition, many determinants of health, such as medical care systems, the social environment, and the physical environment have their biological impact on individuals in part at a population level.” They note that the determinants “include medical care, public health interventions, aspects of the social environment (income, education, employment, social support, culture) and of the physical environment (urban design, clean air and water), genetics and individual behavior.” This is consistent with the description Dunn and Hayes (8) quote from the Canadian Federal/Provincial/Territorial Advisory Committee on Population Health:

“*Population health refers to the health of a population as measured by health status indicators and as influenced by social, economic, and physical environments, personal health practices, individual capacity and coping skills, human biology, early child development, health services. As an approach, population health focuses on the interrelated conditions and factors that influence the health of populations over the life course, identifies systematic variations in their patterns of occurrence, and applies the resulting knowledge to develop and implement policies and actions to improve the health and well-being of those populations.”*

By the nature of these descriptions, population health sciences draw on expertise from basic, clinical, behavioral, and social sciences, providing a rich environment for understanding mechanisms and better defining potential strategies for intervention at various levels of the system. Population health sciences integrate systems science with human development, extending from the individual shaped by their biology and external experiences to that of an individual's contribution to a population.

The proposed definition of population health relevant to health sciences education draws on the above background. In the contest of health sciences education, population health is defined as the health and wellbeing of the individual/patient in context to the population they are a constituent part of. The categories of influence on health are captured within a model that converges disciplines. Linked to this definition are a set of “tools” that permit the exploration of health status, from the individual to the population. This definition supports a pedagogy that reinforces for students the importance of placing the patient in context to the population the patient is part of, whether, for example, the “population” is defined as those with the same condition as the patient or perhaps the community within which the patient lives.

**Figure 1:**
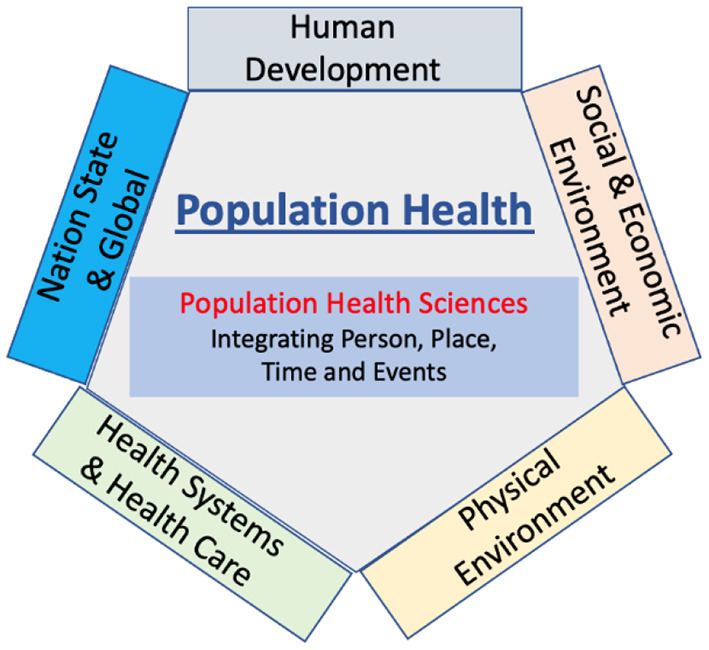
Conceptual model of population health.

The model (Figure 1) starts at an individual level, where health can be viewed as a capacity or resource rather than a state and recognizes the range of social, economic, and environmental influences that contribute to health (15). This level captures our understanding of genetic and biopsychosocial processes of human development, a field that is advancing at a tremendous pace with important implications for health and wellbeing. From pre-conception, through *in-utero* development, to early child development and to development through the life span, our knowledge of individual development is critical to understanding health and wellbeing, disorder, disease, and disability. Within this model students gain an understanding of the life course of an individual, appreciating the key processes involved in human development at an individual level.

The social and economic environment is the second component of the model. Here students gain understanding of the social and economic environment within which individuals live. The units of influence encompass the family, community structures such as employment, education, and recreation that provide a social infrastructure and economic opportunity that supports human development. We now understand better the biological embedding of early experience (16) and how social and economic influence actually “get under the skin” and their impact on biological processes of development and human function (17, 18). The population health framework emphasizes the link between human development and the social/economic structures that influence health and wellbeing. Students will gain a greater understanding of the social and economic structures of their communities and of how these relate to the “life story” of the patient presenting to them with a disorder, disease, or disability.

The third component of the model is the physical environment. The physical environment has influenced human development and health from the earliest of time, whether providing desirable and safe opportunities for settlement, or presenting the challenges of natural disasters, or the challenges of manmade influences on the physical environment. “Where we are born, live, study and work directly influence our health experiences: the air we breathe, the food we eat, the viruses we are exposed to and the health services we can access” (19). Students will gain understanding of the built and natural environments in relation to health, appreciating the importance of “place” and “place across the life-span” (20). Specific areas of importance include the differential impacts of urban and rural environment, climate change, and natural and manmade disasters and health emergencies.

The fourth component of the model is health systems and health care. Elliott et al. (21) define health systems science as an “understanding of how care is delivered, how health professionals work together to deliver that care, and how the health system can improve patient care and health care delivery.” However, from a population health perspective this component of the model is seen as a much broader set of services that support health and wellbeing including promotive and preventative services supported within a public health structure (22–24). This component of the model captures the important evolution occurring in health systems that is driving a greater focus on personal decision making, precision medicine, artificial intelligence and access to data that is transforming how individuals receive information and services that influence their health and wellbeing and access to health care (25–27).

The undergraduate curriculum will include content that builds understanding of health care and the systems delivering care. The model removes the artificial distinction between “health care” and “public health,” providing students with the concepts and knowledge of a continuum of health services (28). Students will have the skills to analyze health systems function, including those directly related to patient care quality and safety in the hospital and ambulatory setting and those related to public health systems (29).

The fifth component of the model is nation state and global. A nation state is defined by a specific geographic boundary within which exists a population and for which the nation state is responsible. The relationship of the “state” to its “people” varies widely but in the end the population within that boundary defines a “state” responsibility. Global health structures and strategies such as the World Health Organization and the United Nations Sustainable Development Goals and many global professional organizations are important, as there are many cross-boundary issues that relate to health and wellbeing. However, it is the “state” that is the ultimate unit of support and responsibility for the health and wellbeing of its population.

Students will gain understanding of the governance structures within a country and the ways in which these structures impact population health. This will include the professional structures and inter-professional relationships that can influence government policies and practices at the local, regional, and national levels. The role of supra-national, global structures in supporting population health will be integrated with the national population health structures.

The sixth component of the model defines the sciences that come together to understand population health—the field of “population health science.” Drawing on multiple disciplines and perspectives, the sciences serve to “integrate” components of person, place, time, and events to better understand how health and wellbeing is achieved. Integration moves from understanding the “individual” to understanding groups of individuals, whether defined by geography, socio-economic conditions, or disorder, disease, or disability. Population health science has a broad range of tools that support exploration through the process of integration. This may be at a biological level measuring for example responses to stressful events, to an individual experience level using qualitative methods, to a broad range of demographic and epidemiological measures that define specific populations of interest, to health systems analytic tools (30) and to the increasing importance of social sciences in complex policy issues (31).

Thus, as captured in Figure 1, population health provides a “convergence” framework for understanding the components that lead to health and wellbeing and to disorder, disease, or disability. The model builds from the individual experience of person, place, time, and events to a set of integrative strategies that allow us to understand, clarify and where necessary influence these components to advance the health and wellbeing of the population. The fields of “public health,” “global health,” “planetary health,” and “health systems” are captured and understood in the context of population health rather than as distinct fields of practice and enquiry and avoids the ongoing attempt to define the distinct nature of these areas (32–37).

For the student, this convergence model provides a clear sense of agency in relation to the needs of their patients and their communities. This supports greater in-country ownership of equity issues and encourages students to see research career opportunities driven by questions within their country and within their control (38), contributing to the health equity goal of “global health” (36).

## Population health in the AKU curriculum

In East Africa, students enter undergraduate medicine from high school with a 6-year curriculum leading to internship and residency. Student selection will be based on academic performance in high school plus a personal interview structure. AKU will primarily draw students from public or private high schools in the East Africa countries of Kenya, Tanzania, and Uganda but admission is not restricted to these countries. AKU has a student financial support structure ensuring that financial capacity will not be a barrier to admission.

Degree programs are accredited in Kenya by the Commission for University Education (https://www.cue.or.ke/) with content guided by the Kenyan Medical Practitioners and Dentists Council (https://kmpdc.go.ke/), the physician licensing authority. Course content and hours are prescribed with flexibility allowed in the structure and mode of delivery.

The AKU curriculum planning has been influenced by the Carnegie Foundation for the Advancement of Teaching (39, 40) and the Lancet Commission on Health Professional Education (41). Education strategies are designed to promote learner-driven acquisition of standardized professional competencies, integration of knowledge with practice, acquisition of a habit of continuous inquiry and improvement, and a strong sense of professional identity.

AKU follows three principles in structuring population health within the undergraduate medical curriculum. First, population health will be delivered as a foundational course with components of the framework embedded in the curriculum, ensuring continuity across courses and years of training. Second, population health would be experiential and laddered in complexity as the student progresses through the curriculum. Third, assessment will capture population health knowledge, attitudes, and practices throughout the curriculum, demonstrating to students the importance of this framework to the overall learning outcomes expected of a professional in the practice of medicine.

The learning outcomes will be achieved through a set of instructional methods that move away from isolated and often disconnected courses to a more integrated and laddered approach to learning and application in context of practice. The following instructional strategies will form the basis of the population health integration.

### Personal perspective and population health

The medical students themselves bring a personal perspective to population health that serves as a learning tool for understanding population health in the context of their life history and development as health professionals. This relationship is supported through a longitudinal mentorship program providing students with a range of opportunities to reflect on their individual characteristics within family and community, their journey as a student in medicine, their understanding and application of professional behaviors, and the balance they achieve between their profession and personal lives.

### Humanities and the arts

The capacity to self-reflect and the skills to capture and be part of a patient's “story” is strengthened through a broad-based education that integrates the arts and humanities within medical education programs (42). This understanding is what has driven increasing recognition of the importance of the arts and humanities in medical education (43). Success as a clinician requires understanding the context of a person's life—their story. Narrative medicine is a common method used in medical education and is defined as “clinical practice fortified with a narrative competence to recognize, absorb, interpret, and honor the stories of self and others” (44). The AKU curriculum will have significant content related to the arts and humanities and this content will be closely aligned with the population health framework.

### Introductory course

A population health foundation course will introduce the AKU population health framework and explore the determinants of health and wellbeing from an individual to a population level. Multiple approaches to delivery will include small group work capturing specific content and the inter-relationship between the components of the model; large group sessions drawing on interdisciplinary expertise; community assessment opportunities using the population health framework; and a range of on-line materials that expose students to key thought leaders and events that illustrate the importance of the determinants and their interaction. The course learning outcomes are listed in Table 1.

**Table 1:** Population health course learning outcomes.

1. Explain concepts and principles related to population health
2. Measure human development and population health outcomes
3. Describe distribution of health across sub-populations
4. Evaluate determinants of health outcomes and the interaction between determinants at various stages of the human life cycle
5. Analyze policies and interventions influencing population health outcomes at the individual and societal level
6. Advocate health education and policy development and interventions to address challenges faced by local communities

### Methodological tools of population health

Students will have a solid grounding in the “integrating tools” of population health and the application of these tool to the clinical cases they work through in the curriculum. Students will understand and apply a range of tools including qualitative methods, demography, statistics, epidemiology, economics, and methods for developing evidence-base and evidence-informed decision making (e.g., systematic reviews). The content will be framed in relation to the model of population health. Students will have the capacity to draw on this base and expand their capacity to use these tools as they move through the curriculum and at increased complexity through the years.

### Case-based learning

The AKU undergraduate medical program will have a strong case-based curriculum. This is ideally suited to including population health content within case-based scenarios (45). Students will explore and bring into discussion the breadth of influences on a given problem within the context of case-based learning objectives. There will be a laddering of the methodological tools that students draw on to understand the case. The learning outcomes related to any given unit will capture important determinants as framed by the population health model. Connecting specific curricular content to learning outcomes (category tagging), will ensure that content related to population health can be captured in the overall curriculum, will be linked to assessment methods, and will contribute to the final learning outcomes. Population health content experts will contribute to case development and assessment and ensure that there is attention to producing a spiraling effect, allowing for demonstrated increased sophistication in use of population health tools for analysis.

### Patient and population

Based on the AKU population health framework the student will “adopt” a patient within a defined geographic population and through a range of experiential opportunities use their clinical skills and the “tools” of population health to understand population health from the “person” to the “population.” Students will have the opportunity to follow a volunteer patient/family gaining experience beyond the patient's clinical problem to the context of the patient's life, providing a “personal” and in-depth understanding of the influences on health and disease. In addition, during an intersession period between Year 2 and Year 3 students will spend time in this geographically defined community setting and develop an applied research project relevant to the population within this region. Practical experience in the community will enable students to bridge the gap between classroom knowledge and the community (38). Students will develop observational and analytic skills as well as skills of communication and collaboration.

### Clinical rotations

Moving from case-based learning to the clinical rotations will provide continuity in framing the understanding of patient care as students now become “responsible” for developing patient history, diagnosis, and management plans. This is where the appreciation for inter-disciplinary work begins to shine—students of medicine, nursing, and other allied health sciences disciplines share in understanding the “patient story” and take responsibility for supporting a therapeutic prescription, a care plan and home/community care plan that can differentiate responsibilities but with a more comprehensive focus on the needs of the patient. In the “best practice” setting, whether in a hospital or a community clinic, there would be an understanding of community structures and resources that can be called on to support patients, “personalizing” the patient experience whether it is a medical intervention, a community social intervention or some combination with “continuity” from primary care to hospital care and back. The use of a population health model provides a common framework and language across disciplines that better support the patient experience.

### Elective planning

Students are encouraged to seek out areas of interest that may align to their future career choices. Given a population health framework, the AKU program will support students in exploring a broader range of elective opportunities. Providing access to high quality electives across the spectrum of determinants, whether local or global will give students an appreciation of the value placed on understanding the breadth and relevance of population health to their professional practice. Expanding the breadth of elective opportunities increases the chance of “capturing” a student's interests that may stimulate career opportunities that move beyond traditional roles.

## Challenges to implementation

Introduction of population health as a foundation structure within an undergraduate medical curriculum must overcome the challenge of abandoning the traditional approach of independent courses, often disconnected from each other. Three key strategies have been used to support the success of this transition.

### Building a Department of Population Health

There are few Departments of Population Health within Medical Schools and there remains territorial resistance to bringing together the various disciplinary components required to provide the foundation of knowledge and skills for success—epidemiology, demography, public health, global and planetary health, health systems sciences, health economics and the social and behavioral sciences. The advantage for AKU is that the “new department” and the “new medical program” will grow together and opportunity to recruit will reduce the disciplinary barriers that established schools may experience. This provides a unique opportunity for a department to work closely with basic sciences and clinical and community medicine in curriculum design and assessment. At the same time, the department will build graduate programs that will align to and support the education and research mission.

### Meeting accreditation requirements

In meeting accreditation requirements, AKU must ensure that the expectations of accreditors can be achieved (46). Through tagging and tracking population health content across the 6 years, it will be possible to translate instructional methods into the content and assessment requirements of the courses defined by the regulators, assuring that content has been covered in the curriculum. This data combined with student and faculty continuous feedback will provide the necessary information to adjust the program as needed and to meet learning outcomes and accreditation expectations.

### Faculty professional development

The success of curriculum reform is determined by both the quality of the reform and the extent to which faculty support change and incorporate change into their teaching. Demands on faculty are significant given the rate of advancements in medicine, the persistent challenge on curricular change, and demands on their professional practice and time for research (47). A population health framework has the capacity to increase efficiency of content delivery while creating a common language that can facilitate faculty support and commitment to the curriculum. Population health provides a framework to start capturing the breadth of health and wellbeing and the many disciplines that contribute to understanding while not compromising depth where this is required. Faculty development will involve a clear blueprint to support this curriculum reform (47). There is faculty support for the population health model as it has efficiency in delivery and captures a cross-discipline commitment to a common model of health and wellbeing.

## Conclusion

The framing of person, place, time, and events is fundamental to our understanding and appreciation of the human experience, to the role a physician has in the “patient story,” and to the physician's potential for impact beyond the individual to that of the population. The understanding and appreciation of “story” links the student's clinical practice to population health and to the value now being placed on integrating arts and humanities content in health professional education.

A Population Health framework supports understanding and enquiry from the cellular to the global, builds appreciation across the disciplines of expertise, and supports a focus on inter-disciplinary/cross-discipline understanding and collaboration. From a policy perspective, health and health care are “local” issues and governments can easily align and appreciate a population health perspective, reducing the tensions between public health and health care priorities, making for more effective policy development, and drawing on a new cohort of physicians who have a better understanding of population health. AKU is committed to evaluation of the model within the delivered curriculum and to measuring the longer-term impact on the practice and career choices of the graduates.

In this paper we present a new approach to framing population health within an undergraduate medical curriculum that we believe will produce graduates with a better understanding of their role and capacity for impact, whether for their individual patient or for the broader community.

The model is applied to the development of a new medical program but has potential application across health sciences and related disciplines, whether established or new.

## Data availability statement

The original contributions presented in the study are included in the article/supplementary material, further inquiries can be directed to the corresponding author.

## Author contributions

Concept development: RA, MM, and AN. Curriculum design, revising the manuscript for critical intellectual content, and decision to submit for publication: RA, MM, AN, LA, and GW. Initial drafting of manuscript: RA. All authors contributed to the article and approved the submitted version.
